# Turning Over an Old Leaf- do Mammalian Herbivores Retain the Ability to Biotransform Toxic Ancestral Diets?

**DOI:** 10.1007/s10886-025-01599-x

**Published:** 2025-04-15

**Authors:** T. J. Orr, M. M. Skopec, S. Kitanovic, K. Y. H. Yamada, Z. Gee, D. Dearing

**Affiliations:** 1 School of Biological Sciences, University of Utah, 257 S 1400 E, Salt Lake City, UT 84112 USA; 2 Department of Zoology, Weber State University, 1415 Edvalson St, Dept. 2505, Ogden, UT 84408 USA; 3 Department of Biological Sciences, Auburn University, 101 Rouse Life Sciences, Auburn, AL 36849 USA; 4 Department of Biology, New Mexico State University, Las Cruces, 88003 USA

**Keywords:** Alpha-pinene, Biotransformation, Diet switching, Herbivory, Mammalian herbivores, Neotoma, Trade-offs, Turnover, Woodrat

## Abstract

Herbivores are frequently exposed to potentially toxic doses of plant secondary metabolites (PSMs). Furthermore, the plant species available and their associated PSMs may change over extended time periods. To understand the ability of herbivores to biotransform novel PSMs, we investigated populations of one species of mammalian herbivore that had undergone a radical diet shift, i.e., the desert woodrat’s (*Neotoma lepida*) switch juniper (*Juniperus* spp) to creosote bush (*Larrea tridentata*). To determine whether woodrats currently feeding on creosote also retain the ability to consume and biotransform the PSMs in their ancestral diet of juniper, we compared various metrics of hepatic biotransformation in a population that ingests creosote bush (Mojave woodrats) to one that specializes on the ancestral diet of juniper (Great Basin woodrats). We investigated PSM biotransformation capabilities by quantifying the hepatic metabolism of α-pinene, a common terpene in juniper. We also measured total cytochrome P450 content, cytochrome P450 2B (CYP2B) and glutathione S-transferase (GST) concentrations, and the activity of GST in the livers of both populations consuming control (rabbit chow) and juniper diets. There were no differences in hepatic metabolism of α-pinene, total P450 content, or CYP2B concentration between woodrat populations when feeding on juniper. The only difference found was that the Mojave woodrats had higher GST activity compared to the Great Basin woodrats when feeding on juniper. Our results suggest that despite the change to a novel toxic diet, the Mojave woodrats maintain the capacity to metabolize their ancestral diet of juniper.

## Introduction

At every meal, mammalian herbivores face the challenge of ingesting potentially toxic plant secondary metabolites (PSMs). Plants produce a vast array of structurally and functionally diverse chemicals to deter herbivory (Mithofer and Boldand 2012). In response, herbivores have evolved a varied array of enzymes to facilitate the ingestion of dietary toxins. These biotransformation enzymes are often tuned ecologically and/or evolutionarily to the metabolism of particular toxins. For example, caterpillars of the butterfly genus *Papilio sp*. specializing on parsnip (*Pastinaca sativa*) have specific detoxification enzymes that allow them to process the PSMs in parsnip more efficiently than generalist caterpillars do (Lee and Berenbaum 1992). Specialization is thought to come at the cost of dietary flexibility because specialists can be less efficient at processing specific toxins that do not typically occur in their diet (Freeland and Janzen 1974). For example, Stephen’s woodrat (*Neotoma stephensi*), a juniper specialist, is unable to process novel toxins (such as phenolics present in creosote) as efficiently as the white-throated woodrat (*N. albigula*), a generalist feeder, but is well-equipped at processing toxins found in its native diet of juniper (Sorensen et al. 2005; Torregrossa et al. 2012). The physiological constraints of efficiently processing dietary toxins suggest there may be trade-offs (e.g., costs) involved in switching from one diet to another, such that the ability to consume the original diet is lost over evolutionary time. Surprisingly, little is known about how a shift to a new diet impacts an herbivore’s ability to process an ancestral diet (Haley et al. 2008). We explored this topic by comparing the biotransformation capabilities of populations of desert woodrats (*Neotoma lepida*) that have shared evolutionary histories, but differ in current dietary profiles.

To better understand the consequences of diet shifts, and the capacity of herbivores to biotransform PSMs experienced only in their evolutionary past, we examined the detoxification abilities of *N. lepida* (the desert woodrat) to feed on its ancestral diet. *Neotoma lepida* is a wide-ranging species in the desert southwest of the USA, inhabiting both the Great Basin and Mojave deserts. This woodrat species evolved in a habitat formerly rich in juniper (*Juniperus* spp.) (Spaulding 1990). However, creosote bush (*Larrea tridentata*) replaced juniper ~ 18 k years ago (KYA) in southern, low elevation areas of *N. lepida*’s range (Hunter et al. 2001; Van Devender 1977; Van Devender and Spaulding 1979; Spaulding 1990; Betancourt 1990). As a result of creosote expansion, many woodrat populations in the Mojave Desert shifted from their ancestral diet of juniper to a novel diet of creosote. Creosote and juniper differ substantially in their PSM profiles. Juniper is rich in monoterpenes, such as α-pinene, sabinene, camphor, and elemol (Adams et al. 1983, 2014, 2016). In contrast, creosote produces large quantities of a resin containing nordihydroguaiaretic acid (NDGA) and hundreds of other compounds, mostly phenolics (Mabry et al. 1977; Hyder et al. 2002). The substantial differences in the PSM profiles of creosote and juniper seem to have selected for new enzymatic machinery to biotransform the diverse and abundant PSMs contained in creosote (Greenhalgh et al. 2022; 2024; Klure et al. 2025; Mangione et al. 2000; Lamb et al. 2004; Haley et al. 2008; Magnanou et al. 2009).

We examined two populations of *N. lepida* that have distinct diets (Skopec et al. 2013 and 2015; Mangione et al. 2000). The Great Basin Desert population (hereafter ‘Great Basin’ woodrats) lacks dietary experience with creosote and feeds on the ancestral diet of Utah juniper (*J. osteosperma*, hereon ‘juniper’), which comprises up to 90% of its diet (Skopec et al. 2015). The Mojave Desert population (hereafter ‘Mojave’ woodrats) currently feeds on creosote bush (*Larrea tridentata*), which comprises up to 75% of its diet (Cameron and Rainey 1972; Meyer and Karasov 1989; Dearing et al. 2022). Previous studies have documented the differences between the Mojave and Great Basin populations with respect to their ability to ingest creosote bush and found that the Mojave population can ingest 25% more creosote resin in feeding trials, compared to the Great Basin population (Mangione et al. 2000). Moreover, these populations differ with respect to the biotransformation enzymes implicated in this process (Mangione et al. 2001, 2000; Lamb et al. 2004; Haley et al. 2008; Magnanou et al. 2009; Malenke et al. 2012, 2014). What remains unknown is whether the Mojave population has retained its ability to biotransform the PSMs in the ancestral diet of juniper.

The goal of this study was to determine whether the Mojave woodrats retain hepatic metabolism of their ancestral diet of juniper in a manner similar to that in the Great Basin woodrats, which currently feed on juniper. We investigated the abilities of both populations to biotransform one terpene (PSM) in juniper, α-pinene (Torregrossa et al. 2011), by comparing their liver metabolism of α-pinene. We quantified the cytochrome P450 content and cytochrome P450 2B (CYP2B) content, as they play a role in the metabolism of terpenes, including those in juniper (Kitanovic et al. 2018a, b; Malenke et al. 2012; Orr et al. 2020; Skopec et al. 2007). We also measured the concentration and activity of glutathione S-transferase (GST) in both populations fed juniper, as it is implicated in the metabolism of creosote bush (Haley et al. 2008; Lamb et al. 2001; Magnanou et al. 2009; Malenke et al. 2014). We addressed two key questions: 1) Do these two populations differ in the rate of metabolism of α-pinene, a key terpene found in juniper? and 2) Do these populations differ in the concentration and activity of key biotransformation enzymes (P450, CYP2B, and GST)?

## Materials and Methods

### Field Sites and Animal Capture

Great Basin woodrats were collected from White Rocks, Tooele County, Utah, USA (40°19’N, 112°53’W). Mojave woodrats were collected from Lytle Ranch, Washington County, Utah, USA (38⁰30’N, 109⁰18’W). Animals were trapped using long Sherman live traps (7.62 × 89 × 22.86 cm). Individuals were transported to the University of Utah Animal Facility where they were housed alone in solid-bottom shoebox cages (48 × 27 × 20 cm) with a layer of shredded aspen bedding (Harlan Teklad) and a plastic tube. The animal facility room was maintained at approximately 24⁰C with an average humidity between 15 and 20%, and a constant light/dark cycle maintained at 12L/12D (methods from Kohl and Dearing 2012; Skopec et al. 2015). Individuals were provided water ad libitum and fed high-fiber rabbit chow prior to feeding trials (Harlan Teklad formula 2031). All procedures had prior approval from the University of Utah Animal Care and Use (IACUC 10–01013 and 16–02011, respectively).

### Experimental Conditions

Animals of both populations were fed either a control diet of rabbit chow or a juniper diet. Rabbit chow is considered the control diet as it is made from plants like alfalfa, soybean, oat, wheat, and corn, which do not produce terpenes (Harlan Teklad formula 2031). Utah juniper (*Juniperus osteosperma*) used in feeding trials was collected from the White Rocks, UT, trapping site. Upon collection, juniper branches with foliage were immediately placed in plastic bags, sealed, and kept in a cooler with ice to maintain volatile compounds. In the lab, juniper was stored at − 20⁰ C prior to diet preparation. Juniper foliage was removed from branches and ground in a Waring blender (model CB- 5) with dry ice until homogenous, and then mixed with ground rabbit chow to prevent sorting and caching by experimental animals (Skopec et al. 2015). Food was presented in feeder hoods (Lab Products Inc.) attached to individual cages to permit accurate measurements of food intake in grams per day. Body mass and food intake were measured at the same time daily. The dry matter of each diet was estimated by placing a known amount of the prepared diet (~ 5 g) in a drying oven for at least 72 h before reweighing. Since we fed diets that had varying amounts of moisture, it was important to calculate dry matter of each diet in order to accurately calculate food intakes.

Four individuals of each population were fed either a control diet without juniper, or an experimental juniper diet presented in a series of increasing concentrations to allow sufficient time for the induction of biotransformation enzymes. Prior to the experiment, animals were maintained on a diet of rabbit chow. During the twelve day experiment, animals on the juniper diet consumed food with each juniper concentration (0%, 20%, 40%, 60% juniper on a dry matter basis) for three days, in succession from the lowest to the highest juniper concentration (days 1–3, 0% juniper; days 4–6, 20% juniper, etc.), while the animals on the control diet were fed ground rabbit chow. On the last day of the feeding trial, animals were anesthetized with isoflurane. Livers were perfused in situ with cold isotonic solution via the hepatic portal vein. Microsomal and cytosolic fractions were prepared by ultracentrifugation as described by Franklin and Estabrook (1971). The microsomal fraction, hereafter referred to as microsomes, contain the endoplasmic reticulum, in which cytochrome P450s are located. Liver samples were stored at − 80 °C. Protein concentrations for all liver samples were determined colorimetrically as described elsewhere (Orr et al. 2020) using Bradford reagent (Sigma) and bovine serum albumin (BSA) standards (Kruger 2002).

### Alpha-Pinene Turnover

We selected α-pinene as our focal substrate because it is a common terpene in Utah juniper (*Juniperus osteosperma*) (Adams et al. 2014).The amount of α-pinene regularly consumed by woodrats causes neurotoxicity, muscous membrane irritation, diuresis, and nephritis in other mammals (Sperling et al. 1967; Savolainen and Pfaffli 1978; Hedenstierna et al. 1983; Falk et al. 1990; Dearing et al. 2000). The metabolism of α-pinene was determined using headspace gas chromatography with flame ionization detection (HS-GC-FID) following methods described in Orr et al. (2020) where the loss of substrate, in this case α-pinene, was an indicator of metabolism. Reactions to assess α-pinene metabolism were conducted in 10 mL glass headspace vials (Agilent), which contained 100 µL of 0.1 M phosphate buffer (pH 7.4), 100 µL 10 mM NADPH, 300 µg prepared microsomes and 10 µL of 20 mM α-pinene in dimethyl sulfoxide (DMSO). Microsomes were allowed to interact with α-pinene (200 µM in DMSO, 0.1 M KPi-buffer, pH 7.4) for a specific period of time (0, 15, and 30 min) at 37 °C. Microsome samples from all individuals were evaluated in duplicate, along with two reaction controls for each time point. Reaction controls were prepared as described above, but without the addition of NADPH as an energy source. All reactions were terminated by placing vials on a hotplate (~ 300⁰C). Quantification of α-pinene was done using headspace gas chromatography with a Tekmar 7000 HT autosampler connected to a Trace GC Ultra with FID (Thermo Scientific). Our HS-GC-FID parameters are fully described in Orr et al. 2020. The HS-GC-FID outputs were manually analyzed in XCalibur (version 2.1.0, Thermo Fisher Scientific), and the area under the curve (AUC) of peaks corresponding to α-pinene were integrated to determine α-pinene concentration. Duplicate values for the AUC for each individual woodrat microsomal sample were averaged to determine the amount of substrate metabolized or percent of α-pinene turnover at each time point. The percent of α-pinene turnover was calculated as follows: [(AUC of α-pinene at time 0 – ACU of α-pinene at 15 or 30 min)/ACU of α-pinene at time 0] * 100. The turnover of α-pinene was analyzed by repeated measures ANOVA with time as the repeated variable, and population and diet as the between-subjects variables. Linear regression was used to determine the relationship between the percent of α-pinene turned over at 15 min and CYP2B content in the statistical software JMP 17. We chose to analyze α-pinene turnover at 15 min, since substrate availability may become limited after 15 min (Orr et al. 2020).

### Determination of P450 Content

P450 content of the microsomal fractions was determined using difference spectrophotometry following Omura and Sato (1964), a method based on quantifying differences in carbon monoxide-binding pigments in liver microsomes. A reduced P450:CO complex occurs in all CYP isozymes and can be quantified by examining absorbance differences in relation to a standard curve (Omura and Sato 1964; Haley et al. 2008). P450 content was analyzed and compared between populations and diet treatments in a two-way ANOVA in JMP 17.

### CYP2B Quantification

CYP2B protein quantification via Western blotting was performed on microsomal fractions thawed on ice and resuspended in 1 M Tris buffer (pH 7.4). After running microsomal liver samples on Mini-Protean TGX Precast Gels (from Bio-Rad), samples were transferred to blots, which were then incubated in a blocking buffer with anti-rat CYP2B rabbit polyclonal antibody (Duignan et al. 1987; Stevens and Halpert 1988) at 1:2000 dilution for 1 h at room temperature. Blots were then washed three times with TBST at room temperature and incubated for 45 min at room temperature with peroxidase-labeled goat anti-rabbit IgG antibody (KPL) in blocking buffer at 1:10,000 dilution. Following three washes in TBST at room temperature, blots were incubated for 5 min in Clarity Western ECL substrate (Bio-Rad) and then imaged with the ChemiDoc MP imager (Bio-Rad).

Bands containing CYP2B were manually selected and further analyzed using ImageLab software (version 5.2.1). CYP2B concentrations (nmol CYP2B/mg of microsomal protein) in liver microsomal samples were estimated based on a standard curve generated from purified woodrat CYP2B37 enzyme (Orr et al. 2020). CYP2B concentration was analyzed and compared between populations and diet treatments in a two-way ANOVA. We also tested for a relationship between liver microsomal P450 and CYP2B concentrations using linear regression in JMP 17.

### GST Content and Activity

GST protein quantification via Western blotting was performed on cytosolic fractions using the same procedures as described for CYP2B quantification, except that cytosol fractions from the liver were used, and blots were incubated with polyclonal goat anti-rat GST-Ya [1:1000 (US Biological)] known to cross-react with human, mouse, and woodrat GST. The blots were visualized with peroxidase-labeled rabbit anti-goat secondary antibodies [1:10,000 (KPL)] and Pierce ECL Western Blotting Substrate (Thermo Scientific). All samples were normalized to a common reference sample run on all gels, as a purified GST standard was not available. GST activity was measured in cytosolic fractions using a GST assay kit (Sigma-Aldrich) with 1-chloro-2,4-dinitrobenzene (CDNB) as a substrate, which absorbs at 340 nm when conjugated with glutathione (Sigma-Aldrich, Habig and Jakoby 1981). The increase in absorbance at 340 nm is directly proportional to the GST activity (Habig and Jakoby 1981). GST content and activity were analyzed and compared between population and diet treatments in two-way ANOVAs. Regression analysis was used to compare the relationship between GST activity and GST content (JMP 17).

## Results

### Evaluation of α-Pinene Metabolism

**Fig. 1:**
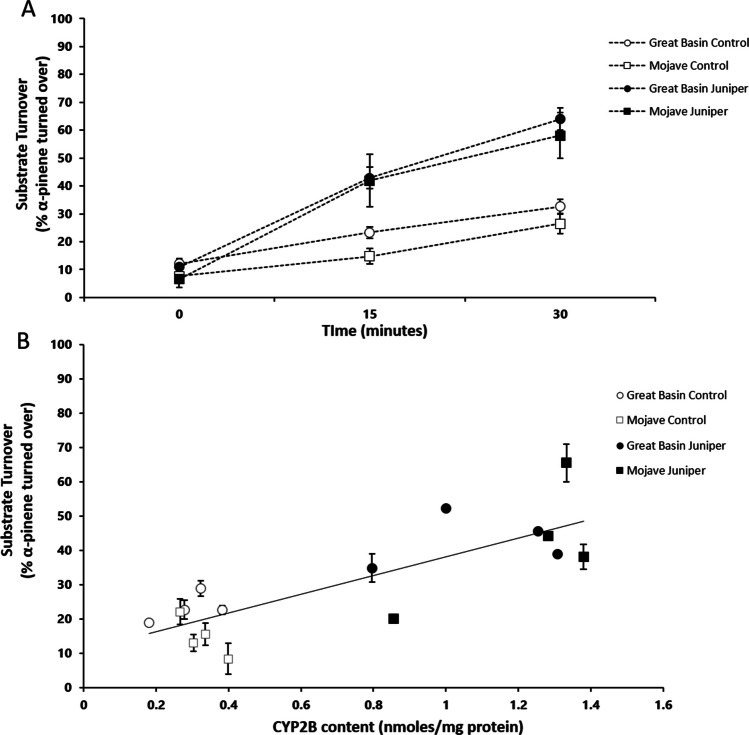
**A)** Microsomal metabolism of α-pinene by reaction time in minutes. **B)** Microsomal metabolism of α-pinene at the 15-min time point against microsomal CYP2B concentration across juniper induction treatments and population. Each point represents the average of the technical replicates for an individual microsomal sample. Bars indicate ± 1SE

**Fig. 2:**
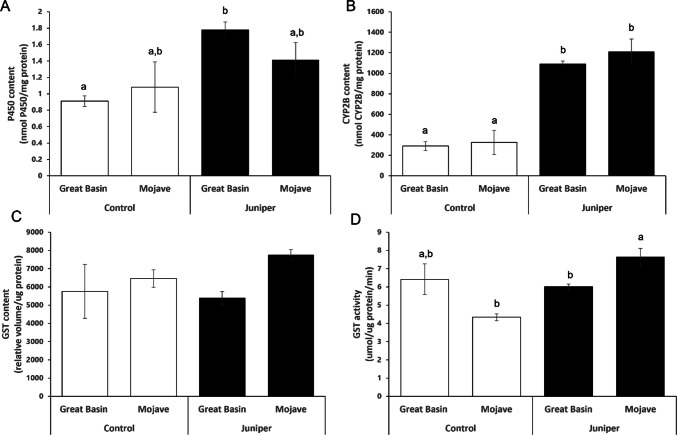
**A)** P450 content **B)** CYP2B concentration **C)** GST content and **D)** GST activity of woodrat livers collected from animals fed control versus 60% juniper diets. Means ± SE are shown. Bars with different letters (a,b) denote means significantly different (p < 0.05) as determined by Tukey’s post hoc tests. No letters are included on Fig. 2 C as the Tukey’s tests did not indicate significance

The microsomes from both populations of woodrats fed juniper had significantly increased α-pinene turnover relative to that in microsomes of woodrats on a control diet. (F_1,12_ = 2.52, p < 0.001, Fig. 1A, 1B). However, there was no significant difference in α-pinene turnover between the populations (F_1,12_ = 0.20, p = 0.14). There was a significant interaction between time and diet (F_2,11_ = 18.62, p = 0.0003), such that α-pinene turnover rates increased at both 15 and 30 min for the juniper-fed animals compared to the controls. A significant amount of variation in α-pinene metabolism rate was explained by CYP2B concentration (R^2^ = 0.66, p = 0.0001, Fig. 2B).

### Woodrat Liver P450 Content and CYP2B Concentration

Juniper feeding induced P450 enzymes in the livers of the Great Basin and Mojave woodrat populations. P450 content was higher in the woodrats fed the juniper diet than in those fed the control diet (mean ± SE: 1.6 ± 0.15 vs. 0.9 ± 0.19 nmol P450/mg protein; F_1,15_ = 12.042, p = 0.005, Fig. 2A); there was no difference in P450 content between the two populations (F_1,15_ = 0.008, p = 0.930), and no significant diet by population interaction (F_1,15_ = 0.947, p = 0.350; Fig. 2A).

**Fig. 3:**
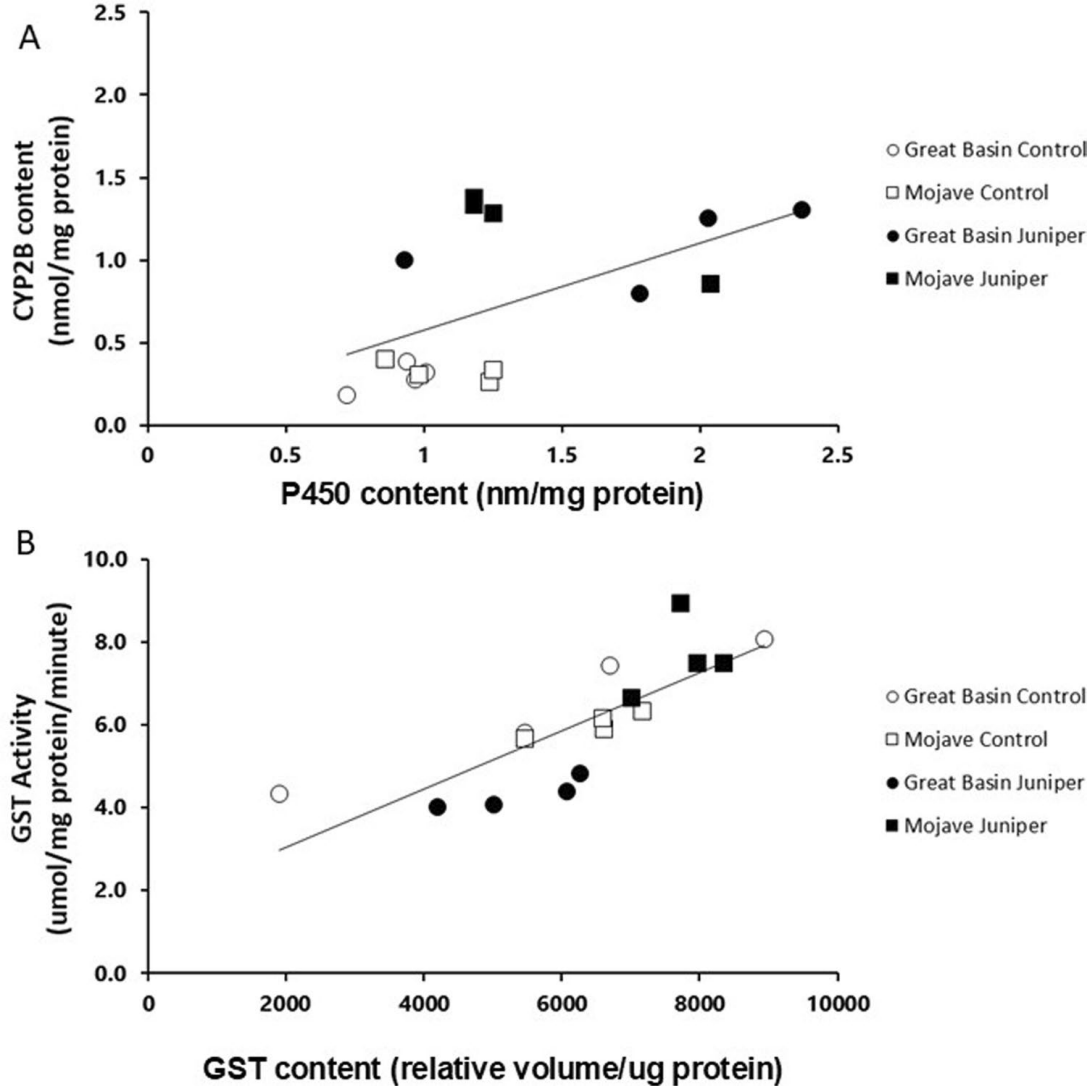
**A)** Microsomal P450 content against microsomal CYP2B content across juniper induction treatments and species. Each point represents the average of the technical replicates for an individual microsomal sample. P450 content explained a significant portion of the variation in CYP2B concentration (R^2^ = 0.31, p = 0.026). **B)** Cytosolic glutathione S-transferase (GST) activity against cytosolic GST content across induction treatments and species. Each point represents the average of the technical replicates for an individual cytosolic sample. A significant amount of GST activity was explained by GST content (R^2^ = 0.64 p = 0.0002)

Juniper ingestion induced CYP2B enzyme concentration in the livers of both woodrat populations. CYP2B concentration was significantly higher in woodrats fed the juniper diet than in those fed the control diet (mean ± SE: 1151.25 ± 74.53 vs. 308.63 ± 80.65 nmoles/mg protein; F_1,15_ = 90.73, p = 0.001, Fig. 2B). CYP2B concentration did not differ between populations (F_1,15_ = 0.790, p = 0.392), and there was no interaction between diet and population (F_1,15_ = 0.246, p = 0.629; Fig. 2B). CYP2B concentration was correlated to overall P450 content (R^2^ = 0.31, p = 0.026, Fig. 3A).

### GST Content and Activity

Though there was a trend of liver GST concentrations differing between the populations (F_1,15_ = 3.62, p = 0.081), they did not significantly differ due to diet (F_1,15_ = 0.34, p = 0.57), and there was no diet-by-population interaction (F_1,15_ = 1.06, p = 0.32, Fig. 2C). Liver GST activity differed between populations, with juniper-fed Mojave woodrats having signficiantly higher GST activity than juniper-fed Great Basin woodrats (F_1,15_ = 6.66, p = 0.026, Fig. 2D). There was also a significant interaction between diet and population (F_1,15_ = 32.11 p = 0.0001), as Mojave woodrats had higher GST activity when fed juniper, while Great Basin woodrats had lower GST activity when fed juniper compared to control diets. Diet did not have a significant effect on GST activity (F_1,15_ = 1.8, p = 0.21; Fig. 2D). GST content was correlated with GST activity (R^2^ = 0.64, p = 0.0002, Fig. 3B).

## Discussion

We investigated whether a population of woodrats that currently feeds on a toxic plant, creosote bush (*Larrea tridentata*), retains the ability to metabolize PSMs present in their ancestral diet of juniper, including α-pinene, a common and toxic terpene found in juniper. In the transition from a juniper diet to a creosote diet (~ 18 KYA), Mojave woodrats appear to have evolved novel physiological mechanisms to deal with the PSMs present in creosote (Dearing et al. 2022; Greenhalgh et al. 2022; 2024; Mangione et al. 2000; Lamb et al. 2004; Haley et al. 2008; Magnanou et al. 2009, 2013; Malenke et al. 2012). This dietary transition may have altered the detoxification pathways of Mojave woodrats used to metabolize the PSMs present in juniper. Curiously, across all our metrics, we found only a single difference between the Mojave and Great Basin woodrats, i.e., differential GST activity in response to a juniper diet. On a diet of 60% juniper, both populations had similar increased P450 content and CYP2B concentrations, and their ability to metabolize α-pinene did not differ. Based on this evidence, we suggest that at least this population of Mojave woodrats has retained their ancestral pathways for the metabolism of the PSMs present in juniper and discuss the implications of this observation below.

### The Role of CYP2B in Juniper and Creosote Feeding

Cytochrome P450s represent a superfamily of biotransformation enzymes that oxidize their substrates, which then undergo either direct excretion, or conjugation followed by excretion (Klaassen and Amdur 2013). Across and even within species, there is a wide diversity of P450 enzymes with multiple isoforms (Gonzalez and Nebert 1990; Guengerich 2008). CYP2B is a subfamily of P450 that plays an important role in the metabolism of terpenes, and therefore has been of interest in the study of juniper specialization of herbivores, including woodrats, for a number of years (see Skopec et al. 2022 for a review). Evolutionary analysis of CYP2B variants in *N. lepida* suggests that CYP2B genes likely evolved in response to dietary terpenes and then were modified in the Mojave populations that switched to creosote (Malenke et al. 2012; Skopec et al. 2022). In woodrats, three of these CYP2B variants (CYP2B35, CYP2B36, and CYP2B37) have been studied for their expression and affinity to α-pinene, as well as their ability to metabolize model substrates (Malenke et al. 2012; Wilderman et al. 2014). When feeding on juniper, both Mojave and Great Basin woodrats express the CY2B35 isoform, while the Great Basin animals also express CYP2B36 (Malenke et al. 2012). When fed creosote, Mojave woodrats express the woodrat-specific isozyme CYP2B37 (Malenke et al. 2012). The CYP2B37 isoform occurs on a novel gene island that is greatly expanded in Mojave woodrats compared to a closely related species *N. bryanti* that also feeds on creosote in the Mojave (Greenhalgh et al. 2022). We do not currently know if Great Basin woodrats also possess the greatly expanded CYP2B island.

The CYP2B antibody used in this study to evaluate CYP2B concentration binds to all CYP2B isoforms, thus we were unable to differentiate expression levels of the different types CYP2B isoforms in this system. As each of these isoforms have different metabolic activity against model substrates, it is possible that they also produce different metabolites of α-pinene and other terpenes. Further study is needed to characterize the differential expression and functional capabilities of the various CYP2B isoforms in woodrats and their impact on juniper consumption and metabolism.

### The Role of GST in Juniper and Creosote Feeding

The only difference between the two studied woodrat populations when fed juniper was greater GST content and activity in Mojave woodrats. GST is a conjugation enzyme that adds a glutathione conjugate onto the products of P450 metabolism (Hayes et al. 2005). The synthesis and excretion of glutathione conjugates are considered energetically costly yet safe for rendering potentially toxic byproducts of P450 metabolism water-soluble and easily excretable (Klaassen and Amdur 2013). GST is rate-limited by glutathione production via the glutamylcysteine synthase enzyme (GCS, Anderson 1998). Wild-caught Mojave animals have considerably higher expression of GCS than wild-caught Great Basin animals (Lamb et al. 2001), thus Mojave animals may be less constrained in their use of GSTs and likely use it as an important step in the metabolism of the phenolics present in creosote. Subsequent studies further support the role of GST in the biotransformation of creosote in the Mojave woodrats (Haley et al. 2008; Magnanou et al. 2009; Malenke et al. 2014). The higher GST activity documented in the Mojave woodrats when feeding on juniper may be the result of their reliance upon a previously evolved detoxification system once used to enable juniper feeding.

Lower reliance on GST may be an adaptation to juniper feeding in the Great Basin woodrats. Another woodrat species that specializes on juniper, *N. stephensi,* downregulates GST when transitioning from a control diet to a juniper diet, similar to the Great Basin woodrats (Skopec et al. 2007; Haley et al. 2007, and herein). It is hypothesized that this is an energy and nitrogen-conserving mechanism, as GST uses a costly glutathione conjugate, which contains three amino acids (Haley et al. 2007). Higher GST activity in Mojave woodrats when feeding on juniper may be needed to modify more reactive metabolites produced by the different CYP2B isoforms in this population compared to juniper specialists. Regardless of the exact cause of the elevated GST activity, this likely results in higher energy and nitrogen requirements in Mojave woodrats compared to the Great Basin woodrats when feeding on juniper. Mojave woodrats may be more impacted during sustained juniper feeding and not able to persist on juniper to the same extent as Great Basin woodrats. Longer-term studies would be necessary to evaluate this hypothesis. For example, another species of woodrat, *N. albigula,* which also upregulates GST when consuming juniper, can only maintain body mass on high-concentration juniper diets for only three days or less (Boyle and Dearing 2003; Haley et al. 2007; Skopec et al. 2007).

### Why do Mojave Woodrats Retain the Ability to Biotransform PSMs from a Diet they Don’t Eat?

There are several possible reasons why Mojave woodrats retain the ability to ingest an ancestral diet of juniper, a diet that is α-pinene-rich. First, the time since divergence of these two populations might not have been long enough for the Mojave woodrats to have lost their ability to metabolize juniper. The Mojave woodrats’ transition from juniper to creosote bush occurred less than 18 KYA, when creosote replaced juniper in the Mojave Desert (Hunter et al. 2001; Van Devender 1977; Van Devender and Spaulding 1979; Spaulding 1990; Betancourt 1990). Moreover, as the Mojave population used in this study inhabits the northern edge of creosote’s range, these animals likely have had less exposure to creosote compared to woodrats in the southern Mojave, therefore additional studies with populations of *N. lepida* that reside in the Southern Mojave desert and have possibly become more specialized to creosote are warranted (Klure et al. 2025). Another possibility is that there may not be a particularly strong selection pressure on the Mojave woodrats to lose their ability to metabolize juniper, if the biotransformation pathways used for juniper are not costly or not used to exclusively metabolize juniper. This lack of selection pressure on less-costly pathways may especially be true of P450s such as the CYP2Bs, which do not utilize valuable conjugates such as GSTs. The degradation of the creosote PSMs by gut microbes in Mojave woodrats may have also relaxed the selection pressure on genes used to biotransform juniper (Dearing and Weinstein 2022; Kohl and Dearing 2012). Lastly, due to individual variation in biotransformation pathways, it is possible that population level differences were not detected in a small sample size of four individuals per treatment. However, previous studies of biotransformation pathways in woodrats have found significant differences between these two populations by evaluating similar sample sizes (Magnanou et al 2009, 2013).

Whatever the mechanism that allows Mojave woodrats to retain their ability to metabolize α-pinene, a major PSM in juniper (Torregrossa et al. 2011), a resulting major benefit would be dietary flexibility. *Neotoma lepida* has one of the largest distributional ranges for a woodrat species and is considered a facultative specialist that can specialize on a variety of foods in different habitats (Skopec et al. 2015). As discussed here, different populations of *N. lepida* specialize on different plants, such as juniper and creosote, but also on mesquite and cactus in other parts of their range (Smith et al. 2014; Brown et al. 1972; MacMillen 1964; Weinstein et al. 2021). An array of mechanisms to metabolize PSMs present in different classes of plants may have allowed for *N. lepida’s* dietary flexibility leading to its range expansion.

## Data Availability

Data will be made publicly available on Dryad upon publication of this manuscript.
